# Otolaryngology Match 2020-21: Survey of Prospective Applicants in the Setting of COVID-19

**DOI:** 10.1177/0003489420952470

**Published:** 2020-08-19

**Authors:** Said Izreig, Sina J. Torabi, David A. Kasle, Rahmatullah W. Rahmati, R. Peter Manes

**Affiliations:** 1 Department of Surgery (Division of Otolaryngology), Yale University School of Medicine, New Haven, CT, USA

**Keywords:** survey, 2021 match, otolaryngology applications, COVID-19, virtual Interviews

## Abstract

**Objectives:**

To capture the perspectives of candidates applying for otolaryngology residency positions in the 2020-21 cycle, in the context of disruption caused by the coronavirus disease 2019 (COVID-19) pandemic.

**Subjects and methods:**

Candidates planning to apply to the otolaryngology 2020-21 match were invited to complete a cross-sectional online survey. Distribution was via otomatch.com and word of mouth. Descriptive statistics were performed.

**Results:**

Of 85 eligible responses (estimated 18.9% of all applicants), many have had at least one board examination (71.8%) disrupted. A majority (85.9%) believe evaluation of candidates will change due to the pandemic, and 54.1% report they were now less confident in matching. Female applicants (37.6% of respondents) were found to have significantly higher odds of decreased confidence in matching (OR 2.781 [95% CI 1.045-7.4044]; *P* = .041). Many report a move to virtual interviews would increase the number of applications submitted (45.9%) and the number of interviews attended (77.6%). Some applicants (36.5%) did not believe residency programs would gather sufficient information about their candidacy to make an informed decision, and most (62.4%) did not believe that they would gather sufficient information to inform their own rank list.

**Conclusions:**

We find that candidates believe their candidacy will be assessed differently in light of the COVID-19 pandemic, are largely less confident in successfully matching, and are planning to apply and interview more broadly. These data are relevant to otolaryngology residency leadership to inform clear dialogue and a smooth transition into an unprecedented application cycle.

## Introduction

SARS-CoV-2, the causative viral agent of coronavirus disease 2019 (COVID-19), has rapidly spread globally since its initial discovery in Wuhan, China in December 2019.^1^ A global pandemic was declared in March 2020, motivating continued restrictions globally on social engagement and travel in order to minimize virus transmission. As medical resources have realigned toward the care of the COVID-19 patient, the provision of medical care for non-COVID patients has also changed.^2^ These changes have included increased usage of telemedicine and moratoriums on elective surgery in order to minimize viral spread.^2,3^ While prudent for minimizing viral exposure, these adjustments carry downstream effects, notably including disruptions to medical education. The Association of American Medical Colleges (AAMC) has recommended that students not be expected to engage in clinical work without adequate safety precautions.^4^ The consequences of these disruptions on the class of 2021 and beyond, however, are not yet clear.

Otolaryngology – head and neck surgery (OTO-HNS) has historically been among the most competitive specialties, with a match rate of 69.3% in 2019-20.^5^ Metrics used to gauge applicant competitiveness, such as average number of research activities and United States Medical Licensing Examination (USMLE) scores, have increased over time.^6,7^ With disruptions driven by the pandemic, prospective OTO-HNS candidates are faced with unprecedented challenges. In light of recommendations for suspension of away rotations for students with a home OTO-HNS program, letters of recommendation to be from local faculty, and residency interviews being conducted virtually, both residency programs and applicants are navigating the coming application cycle with less shared information and more uncertainty.^8,9^

Given the unprecedented nature of the disruptions of COVID-19 on medical education and residency applications, a full account of the repercussions of the pandemic on graduating medical students remains an outstanding issue. To assess the impact of the COVID-19 pandemic on prospective OTO-HNS candidates, we conducted a cross-sectional survey of applicants planning, or have planned, to apply to OTO-HNS in the 2020-21 residency application cycle. Our survey was designed to capture COVID-19-related disruptions, as well as perspectives regarding the coming application cycle. Finally, we aimed to collect outstanding concerns applicants wish to have addressed. Our objective was to better inform stakeholders in the upcoming OTO-HNS match of the challenges faced by prospective otolaryngologists.

## Methods

### Survey Creation and Content

The Yale Qualtrics Survey Tool (Qualtrics, Provo, UT) was used to create a cross-sectional survey shown in Supplemental Figure 1. Our target population included students or recent graduates planning on applying into OTO-HNS in the 2020-21 application cycle, or those who at some point considering applying but opted to delay graduation (e.g. to perform research). Respondents who did not match the above profile were not able to continue with the survey. As the survey was anonymous, the Yale Human Investigations Committee determined this study to be exempt from review.

The survey consisted of a maximum of 23 questions estimated to take 7.5 minutes to complete. The majority were multiple choice, though four required respondents write free text, and six allowed text if respondents chose to expand. In brief, we obtained demographic data (graduation year, gender, attendance at a top 40 NIH-funded medical school, home OTO-HNS program availability, and whether respondents were taking a research year), how the pandemic has affected their medical education (core clerkships and USMLE exams), and how time has been utilized during the pandemic. We queried respondents on their beliefs regarding whether the pandemic will affect the application cycle, and about their opinions on virtual interviews.

### Survey Dissemination

The survey was principally disseminated via Otomatch.com and was posted May 18th, 2020. The survey was also disseminated via e-mail through the AAMC Organization of Student Representatives on May 21st, 2020, where student representatives were asked to forward invitations to their OTO-HNS interest groups. As incentive, respondents who completed the survey were linked to a second survey which had the option to input an e-mail address to enter a raffle for one of two $50 Amazon gift cards (dispensed on May 27th, 2020). We required a medical school-affiliated e-mail to prevent ballot stuffing. A second survey was used for this purpose to maintain anonymity. The survey was closed on May 25th, 2020 owing to slowed recruitment with no other means of dissemination.

### Survey Analysis

We provide descriptive analyses on each question. After survey completion free text responses were thematically categorized during analysis by two authors (S.I. and S.J.T.), and disagreements were resolved through discussion and mutual agreement All free text responses and thematic categorizations are detailed in Supplemental Figure 2. We stratified data based on demographics, and performed chi-square or Fisher’s Exact Test, as appropriate, to elucidate differences in confidence in matching based on demographics. A single multivariate binary regression analyzing confidence in matching was performed utilizing all variables with a p<0.500 on univariate analysis. Statistical significance was defined as a p-value of ≤0.05. Figures were created via GraphPad Prism v8 (GraphPad Software, San Diego, CA).

## Results

### Survey Population and Demographics

One hundred fifteen individuals advanced past the introductory page. Of these, 87 (75.7%) completed the survey. The remaining respondents either did not finish or indicated that they never planned to apply in the upcoming cycle. Due to issues with power, we excluded two respondents who indicated they would delay application by a year (neither due to COVID-19). With a conservative assumption of 450 applicants in the upcoming cycle, based on an average of 433 from the previous three application cycles, 85 responses reflects approximately 19% of predicted applicants.

Of 85 participants, 32 (37.6%) were female, 62 (72.9%) were planning on graduating within four years, 35 (41.2%) attended a top 40 NIH-funded school, and 82.4% attended a medical school with an affiliated OTO-HNS residency program (Table 1).

**Table 1.** Survey Responder Demographics.

	All Participants (n = 85)
**Gender**	
Male	53 (62.4%)
Female	32 (37.6%)
**Original Graduation Year**	
Class of 2021 (ie, graduating in 4 years)	62 (72.9%)
Class of 2020 (ie, taking a 1 year leave of absence or research)	16 (18.8%)
MD/PHD^A^	2 (2.4%)
Reapplicant (from any original graduation year)^A^	4 (4.7%)
Multiple (>1) Research Years^A^	1 (1.2%)
**Attendance at a Top 40 NIH-Funded School**	
Yes	35 (41.2%)
No	49 (57.6%)
Preferred to not answer	1 (1.2%)
**Presence of a Home ENT Residency Program**	
Yes	70 (82.4%)
No	15 (17.6%)

A Variable built from free text response, in response to “other” (Supplemental Figure 2).

### Applicant Activity and Curriculum Disruptions during the COVID-19 Pandemic

The most common activities by applicants during the pandemic has been research (92.9%), followed hobbies/personal development (74.1%), and family time (63.5%). Few applicants report being directly affected by COVID-19 (4.7%) (Supplemental Figure 3).

**Figure 1.**
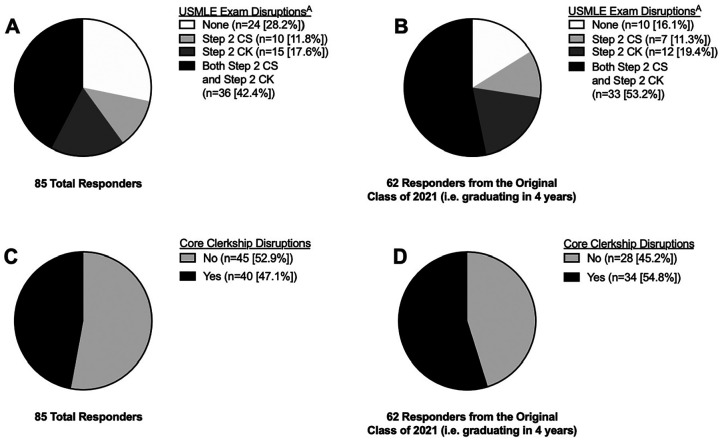
Pandemic disruptions for A) USMLE examinations for all, B) USMLE examinations for original class of 2021, C) core clerkships for all, B) core clerkships for original class of 2021. ^A^No Step1 disruptions.

With regards to USMLE exams, 71.8% of respondents indicated that either Step 2 CS and/or CK have been disrupted due to the pandemic (Figure 1A), which increased to 83.8% when including only those who were in the entering class of 2021 (ie, graduating in four years) (Figure 1B). With regards to core clerkships, 47.1% indicated some sort of disruption to completion of clerkships (Figure 1C), which increases to 54.8% of the entering class of 2021 (Figure 1D).

### Consideration for Research Years

**Figure 2.**
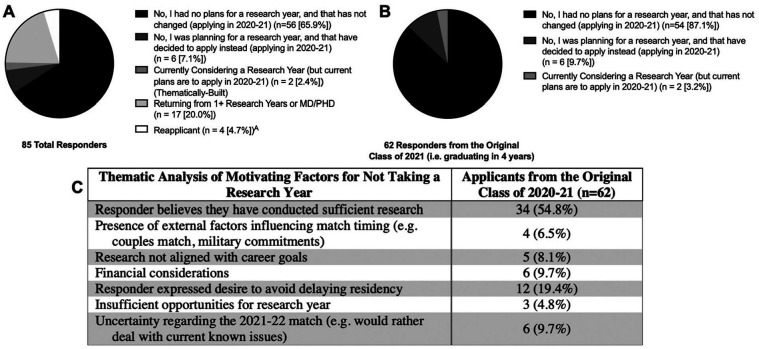
Research year plans in A) all responders and B) those from original class of 2021. C) Thematic analysis of motives for not taking a research year. ^A^Respondents disclosed reapplicant status elsewhere in survey.

Twenty percent of our cohort were returning from research years, and there were no applicants who indicated that they were planning on taking a research year in large part due to the COVID-19 pandemic (Figure 2A). Of the graduating class of 2021, two (3.2%) indicated that they were currently considering a research year, and another six (9.7%) stated that they were planning on a research year but decided to apply instead (Figure 2B). Thematic analysis of reasons for deciding against a research year among the graduating class of 2021 revealed that 34 (54.8%) believed they had sufficient research and 12 (19.4%) expressed a desire to avoid delaying residency. Less commonly cited reasons included external factors influencing match timing (e.g. couples matching) (6.5%), financial considerations (9.7%), uncertainty regarding the 2021-22 match (9.7%), and research not aligning with career goals (8.1%) (Figure 2C).

### State of Otolaryngology Training During the COVID-19 Pandemic

The majority of applicants, excluding six who already completed a sub-internship, indicated that they will either definitely or likely be able to complete at least one sub-internship (86.1%), but three individuals (3.8%) indicated they will likely or definitely not be able to complete a sub-internship (data not shown [dns]). However, a larger fraction indicated that they were unsure about whether they will have acquired the clinical exposure and training required to function as an intern in OTO-HNS by graduation (31.8% of all 85 responders and 37.1% of those in the entering class of 2021; dns).

### Beliefs Surrounding Possible Changes in Applicant Evaluations by Programs

**Figure 3.**
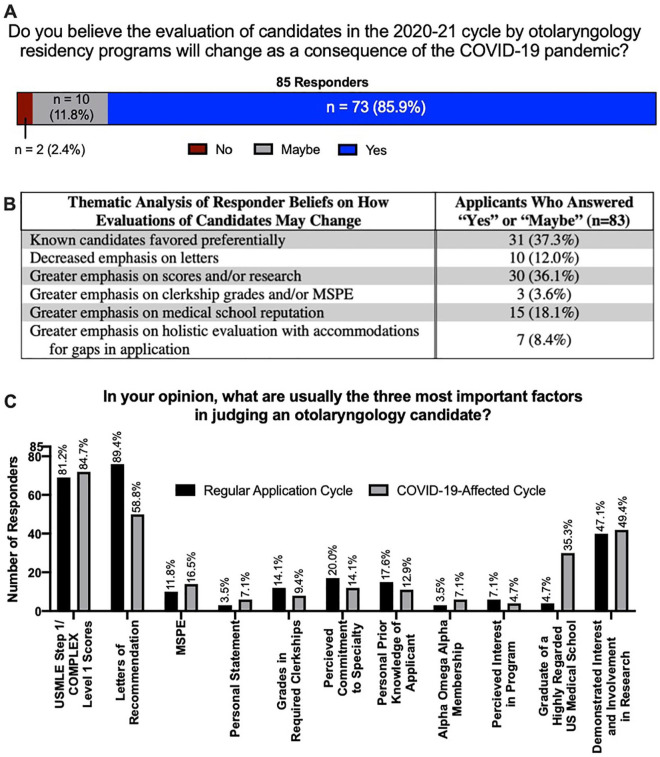
A) Responder beliefs regarding changes in candidate evaluation, B) thematic analysis of responder beliefs, and C) applicant beliefs regarding top three factors in judging an applicant pre- and post-pandemic.

Using evaluation criteria included in the National Residency Match Program survey as a guide,^10^ the majority of responders, 73 (85.9%), believe that evaluation of candidates will change as a result of the pandemic (Figure 3A). In thematic analysis of free text, 37.3% believed that candidates known to programs would be favored and 36.1% believed there will be greater emphasis on scores and/or research. Only 7 individuals (8.4%) reported that they believed there would be more holistic evaluation with accommodations for gaps in applications (Figure 3B). When asked directly about the three most important factors during a normal application cycle, 81.2% indicated USMLE Step 1 scores, 89.4% letters of recommendation, and 47.1% interest and involvement in research. Comparatively, in a COVID-19-affected cycle, applicants similarly endorsed the importance of step 1 scores (84.7%) and research (49.4%). However, while letters of recommendation still received the third highest number of votes (58.8%), graduating from a highly regarded medical school increased from 4.7% in a regular cycle to 35.3% in a COVID-19-affect cycle (Figure 3C).

When applicants were asked to detail the types of candidates believed to be at a relative disadvantage this cycle, 78.8% indicated that those with no home programs or limited mentorship, and 22.4% indicated that those who were relying on Step 2 CK or away rotations to make up for a perceived weakness (Table 2).

**Table 2.** Applicant Beliefs on Candidates Who may be Disadvantaged.

Thematic Analysis of Responder Beliefs on Candidates that May be Disadvantaged	Number of Responders (n = 85)
Students with no home program or limited mentorship opportunity	67 (78.8%)
Graduate of an osteopathic (DO) institution	7 (8.2%)
Candidates relying on Step 2 CK or away rotations to bolster their applications or make up for a perceived weakness	19 (22.4%)
Candidates with regional or institutional preference	5 (5.9%)
Candidates with a late interest in ENT	8 (9.4%)

### Confidence in Matching

**Figure 4.**
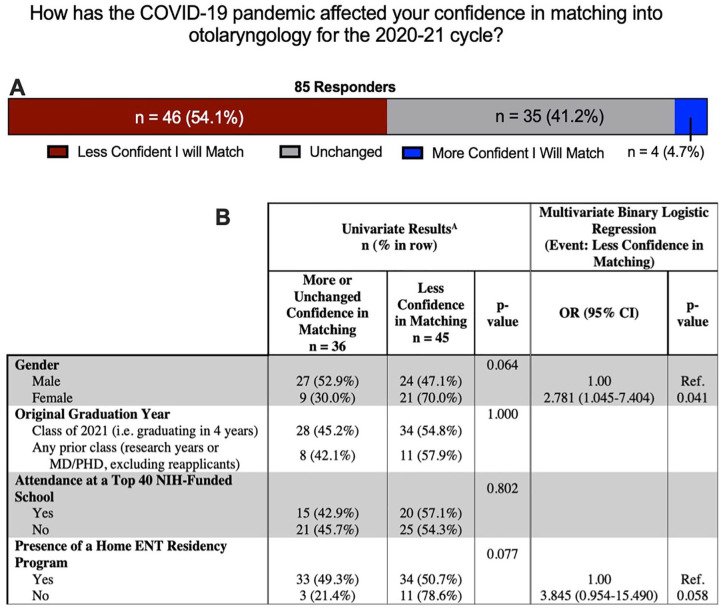
(A) The effect of the COVID-19 pandemic on responder confidence, and (B) variations with respect to demographics. ^A^Excluding four re-applicants.

The majority of responders (54.1%) reported that they were less confident in matching this year due to the COVID-19 pandemic, with another 41.2% indicating that their confidence was unchanged. Only four individuals (4.7%) reported increased confidence (Figure 4A). When comparing demographics between those with more or unchanged confidence vs less confidence, we noted that 70.0% of female applicants were less confident in matching, more than the 47.1% of male applicants, though not significant (*P* = .064) (Figure 4B). No difference was noted in graduation year or attendance of a top-40 NIH-funded school (*P* > .05 for both), but more individuals without home ENT programs reported decreased confidence (78.6% vs 50.7%), though not significant (*P* = 0.077). In a multivariable binary logistic regression, female applicants had statistically higher odds of reporting decreased confidence in matching (OR 2.781 [95% CI 1.045-7.4044]; *P* = 0.041). Presence of a home OTO-HNS program also carried increased odds of decreased confidence, though not significant (OR 3.845 [95% CI 0.954-15.490]; *P* = 0.058). More variables were not included to prevent over-fitting.

### The Effect of Virtual Interviews on Application Patterns

**Figure 5.**
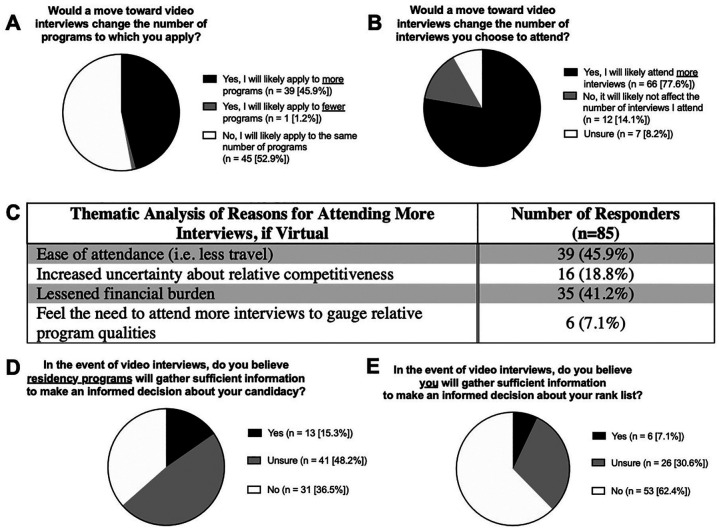
(A) Virtual interview effect on application number and (B) interviews attended, (C) reasons for attending more virtual interviews, (D) responder beliefs on informed decisions by programs, and (E) by applicants.

A majority of applicants stated that they would not apply to more programs (52.9%), though a sizeable fraction indicated they would (45.9%) (Figure 5A). In contrast, the majority of respondents indicated that they would attend more interviews (77.6%) (Figure 5B). Reasons cited for attending more interviews, when thematically grouped, included ease of attendance (eg, less travel) (45.9%), decreased financial burden (41.2%), uncertainty about relative competitiveness (18.8%), and need to attend more interviews to gauge relative program qualities (7.1%) (Figure 5C). Seven of 12 responders who reported they would not interview more than usual stated they were planning on interviewing broadly regardless (Figure 5B).

Thirty-one (36.5%) applicants did not believe residency programs would gather sufficient information to make informed decisions about their candidacy (Figure 5D). Fifty-three (62.4%) applicants did not believe they would gather sufficient information to make informed decisions about their own rank list (Figure 5E).

When asked about preferences in virtual interviews, nearly all respondents expressed interest in informal chats with residents (98.8%), program director presentations/Q&A (88.2%) and virtual facility tour (84.7%) (Supplemental Figure 4).

### Other Questions and Concerns

Twenty-six individuals (30.6%) expressed other concerns not captured by the survey, (Supplemental Figure 2). Though broad in scope, seven (26.9%) requested insight into how programs are planning to evaluate candidates in light of the COVID-19 pandemic, seven (26.9%) requested information regarding interview structure, and three (11.5%) requested guidance on how best to express interest in particular programs.

## Discussion

The COVID-19 pandemic has demanded rapid adjustment at all levels, social and professional, to limit the hazard to human health. Medical education has not been spared. In light of national guidance recommending virtual interviews for the coming residency application cycle, and the discouragement of away rotations except for select cases, the landscape for residency applicants and programs has drastically changed.^4,8^ Herein we present a survey of prospective OTO-HNS applicants to better inform all parties of the concerns and challenges faced by candidates.

Within our diverse sample of 85 applicants (approximately 1 in 5 estimated applicants), an overwhelming majority (92.9%) reported engaging in research during the COVID-19 pandemic (Supplemental Figure 3). This is in agreement with trends of increasing research output of OTO-HNS applicants over time, suggesting that applicants have identified research productivity as a key component of their relative competitiveness.^11^ Given previous reports of increased rates of research years undertaken by OTO-HNS applicants,^6^ we hoped to address whether the COVID-19 pandemic influenced applicant preference towards delaying graduation for a research year. Strikingly, no respondents reported changing plans from graduating in 2021 after four years to delaying graduation for research (Figure 2), although it is possible such students may have not been captured by our survey. Instead, 9.7% of respondents reported cancelling a planned research year and graduating in four years. Reasons cited for this reversal included “perceived competitiveness of the following cycle,” “inability to relocate to place of research,” and “additional time with delay of ERAS,” implying added productivity during COVID-19-related disruptions (Supplemental Figure 2). Thematic analysis suggests that a majority of applicants (54.8%) believe they have sufficient research to be competitive. These results suggest that the COVID-19 pandemic did not disrupt most research activity, though a minority did describe irreconcilable research disruptions (Supplemental Figure 2).

A common theme pervading our survey is one of diminished confidence among applicants approaching a COVID-19-affected cycle. This is reflected in applicant assessments about their likelihood of matching, where a majority (54.1%) stated they were less confident they will match in the 2020-21 cycle (Figure 4). Notably, female gender carried significantly higher odds of reporting lower confidence matching in light of COVID-19 disruptions (OR 2.781 [95% CI 1.045-7.4044]; *P* = 0.041) (Figure 4). Though beyond the scope of this study to identify reasons for this disparity, women are still underrepresented in the OTO-HNS work force.^12^ These data emphasize that efforts to present OTO-HNS as a welcoming specialty to all genders remains important in fostering continued interest in the field.

A potential contributor to applicant uncertainty may relate to the majority belief (85.9%) that candidate evaluation will change in the upcoming cycle (Figure 3A). Interestingly, 35.3% identified graduating from a highly regarded US medical school as one of the top three factors candidates will be evaluated by in the coming cycle, whereas only 4.7% believed this to be the case in a regular cycle (Figure 3C). In 2018, the fraction of matched applicants to otolaryngology from top-40 NIH funded schools (30.1%) was similar to the fraction of matched applicants across all specialties (31.9%),^13^ suggesting medical school of origin was not as important as individual qualifications in a regular OTO-HNS cycle. Whether this observation remains true for the coming cycle, however, is unknown.

Respondent beliefs regarding who may be preferentially disadvantaged reflect the perceived importance the home institution will have on an applicant’s success, as 78.8% of respondents believe those applicants without a home OTO-HNS program are particularly disadvantaged (Table 2). Given that perceived “reputation” and available resources of their medical school is outside the immediate control of applicants, a perceived shift toward greater emphasis on school affiliation may be contributing to diminished confidence among applicants. To the degree residency programs can provide insight regarding their approach to candidate evaluation, such as typical profiles of historically interviewed candidates, this may bolster the confidence among applicants facing a particularly uncertain year.

As OTO-HNS programs navigate the logistics of preparing for virtual interviews, our results show respondents anticipate changing their approach to the application and interview season. While only 45.9% of respondents predict applying to more programs, 77.6% believe they will attend more interviews in light of the virtual format (Figure 5A,B). In addition, respondents do not believe (36.5%) or are unsure (48.2%) that residency programs will gather sufficient information to accurately gauge their candidacy (Figure 5D). With less confidence in being able to project their candidacy effectively, respondents are suggesting they will interview as broadly as possible to offset the perceived disadvantage.

Previously, the percentage of applicants ranking ≥16 programs was 26.6% in 2016, which increased to 35.4% in 2018.^13^ Given that traditional barriers to number of attended interviews – costs and temporospatial restrictions – are absent in the coming cycle, it is conceivable this figure will increase. It has also been reported that 26% of OTO-HNS applicants attended 50% of interviews, suggesting that OTO-HNS programs were vying for a smaller pool of upper echelon of candidates.^14^ Without external constraints, the competition for top candidates is likely to intensify. This arrangement risks unfilled programs, unmatched applicants, and less-than-ideal matches as applicant preference is unable to be determined.^15^ In addition, OTO-HNS programs are already facing challenges processing applicant volume.^16^ This dynamic presents a challenge for both OTO-HNS programs and applicants in the interview and ranking process. Whether programs decide to simply interview more applicants or employ alternative solutions, such as preference signaling, is yet to be determined.^17,18^ While other specialties have encouraged applicants to attend fewer interviews, compliance may be an issue without mechanisms of enforcement.^19-21^

The major limitation of our study is response bias. It is possible we captured only those motivated to respond. Likewise, we may have captured a sample not representative of all applicants. However, this is partially mitigated by relatively clear consensus along some dimensions of our survey. We were similarly limited by answer choices. Though we attempted to mitigate this by thematically grouping free text responses, the nature of free text prevents assessing how many participants believe in a thematic option. For example, that 78.8% report they believe those without home programs are disadvantaged does not mean that 21.2% do not. This similarly holds true for thematic groupings with small sample sizes, such as 2.4% of applicants expressing interest in learning about local culture during virtual interviews.

## Conclusion

The challenges imposed on the coming residency application cycle by the COVID-19 pandemic are exacerbated by a lack of shared information among applicants and programs. Our survey of prospective OTO-HNS candidates offers insight into the challenges they face, along with prospective guidance regarding their application strategies. Our results suggest fostering clear communication with applicants to address underlying uncertainty regarding candidate evaluation and the interview process would be well received.

## Supplemental Material

Supplemental_Figure_1 – Supplemental material for Otolaryngology Match 2020-21: Survey of Prospective Applicants in the Setting of COVID-19 Click here for additional data file. Supplemental material, Supplemental_Figure_1 for Otolaryngology Match 2020-21: Survey of Prospective Applicants in the Setting of COVID-19 by Said Izreig, Sina J. Torabi, David A. Kasle, Rahmatullah W. Rahmati and R. Peter Manes in Annals of Otology, Rhinology & Laryngology

Supplemental_Figure_2_-_Revised – Supplemental material for Otolaryngology Match 2020-21: Survey of Prospective Applicants in the Setting of COVID-19 Click here for additional data file. Supplemental material, Supplemental_Figure_2_-_Revised for Otolaryngology Match 2020-21: Survey of Prospective Applicants in the Setting of COVID-19 by Said Izreig, Sina J. Torabi, David A. Kasle, Rahmatullah W. Rahmati and R. Peter Manes in Annals of Otology, Rhinology & Laryngology

Supplementary_Figure_3 – Supplemental material for Otolaryngology Match 2020-21: Survey of Prospective Applicants in the Setting of COVID-19 Click here for additional data file. Supplemental material, Supplementary_Figure_3 for Otolaryngology Match 2020-21: Survey of Prospective Applicants in the Setting of COVID-19 by Said Izreig, Sina J. Torabi, David A. Kasle, Rahmatullah W. Rahmati and R. Peter Manes in Annals of Otology, Rhinology & Laryngology

Supplementary_Figure_4 – Supplemental material for Otolaryngology Match 2020-21: Survey of Prospective Applicants in the Setting of COVID-19 Click here for additional data file. Supplemental material, Supplementary_Figure_4 for Otolaryngology Match 2020-21: Survey of Prospective Applicants in the Setting of COVID-19 by Said Izreig, Sina J. Torabi, David A. Kasle, Rahmatullah W. Rahmati and R. Peter Manes in Annals of Otology, Rhinology & Laryngology
