# Supplementary material for: Otolaryngology Match 2020-21: Survey of Prospective Applicants in the Setting of COVID-19
Source: Ann Otol Rhinol Laryngol. 2020 Aug 19;130(5):450–8. doi: 10.1177/0003489420952470 (PMC7481654; doi:10.1177/0003489420952470)
Supplement: Supplemental_Figure_1 – Supplemental material for Otolaryngology Match 2020-21: Survey of Prospective Applicants in the Setting of COVID-19 [file Supplemental_Figure_1.pdf]

Appendix A. Full Survey

ENT Applicant Survey 2020-21

Start of Block: Applying Question

INTRO
Impact of COVID-19 on the Otolaryngology Application Cycle
A 5-Minute Survey

IRB#2000028179

Dear Otolaryngology Candidate,

We are conducting an anonymous survey regarding the impact of COVID-19 on the Otolaryngology application process for the 2020-21 application cycle. Our goal is to better understand the perspective of the ENT-interested medical student given the unprecedented uncertainty. The results we obtain will be shared with Otolaryngology programs nationally in order to better inform all involved parties.

The full survey consent form may be found as a link at the bottom of this survey. In addition, the exemption is listed below. However, in brief, your participation is voluntary. You are not obliged to answer questions to which you object to or do not find appropriate. All answers provided are confidential and participant identities are anonymized. We do not anticipate any risks from participating in this study. Your participation in this study implies informed consent.

Below is a link to the survey which should take no more than 5-10 minutes to complete. Your input is greatly appreciated and will contribute to easing the uncertainties of the coming year.

Pending recruitment, we anticipate closing the study by July 2020.

If you have any questions about the survey or study, please feel free to contact us at david.kasle@yale.edu or rpeter.manes@yale.edu.

Thank you for your participation,

Dr. David Kasle,
PGY-4, Yale Otolaryngology

Dr. R. Peter Manes

Rhinology and Endoscopic Skull Base Surgery
Associate Professor
Associate Residency Program Director
Division of Otolaryngology
Yale School of Medicine

[Online consent]

[IRB Exemption]

End of Block: Applying Question

Start of Block: 1. YES/NO

Q1 Were you previously, or are you currently planning to apply to an otolaryngology residency position in the 2020-21 application cycle?

- ◯ Yes
- ◯ No

End of Block: 1. YES/NO

Start of Block: 2. Applicant Information

Q2 What was your original medical school graduation year, upon matriculation?

- ◯ Class of 2021 (i.e. graduate in 4 years)
- ◯ Class of 2020 (i.e. took a 1 year leave of absence or a research year)
- ◯ Other (MD/PhD, joint degree, etc.) ____

Q3 What is your gender?

- ◯ Male
- ◯ Female
- ◯ Other ____

Q4 Do you attend a Top 40 NIH-Funded medical school?

(List may be found here: https://www.genengnews.com/a-lists/top-50-nih-funded-institutions-of-2019/)

- ◯ Yes
- ◯ No
- ◯ Prefer to not answer

Q5 Does your home medical institution have an otolaryngology residency program?

- ◯ Yes
- ◯ No

Q6 How has your time been spent during the COVID-19 pandemic? Please select all that apply.

- ☐ Clinical Activities
- ☐ Research
- ☐ Volunteering
- ☐ Hobbies/Personal Development
- ☐ Dedicated Study Periods for USMLE exams
- ☐ Studying for a non-USMLE subject
- ☐ Recovering from suspected or confirmed COVID-19
- ☐ Helping a family member recover from suspected or confirmed COVID-19
- ☐ Family time
- ☐ Other ____

Q7 For which of the following USMLE examinations has the COVID-19 pandemic affected your examination plans? Please select all that apply.

- ☐ None
- ☐ Step 1
- ☐ Step 2CS
- ☐ Step 2CK

Q8 Has the COVID-19 pandemic affected your ability to complete your core, non-elective clerkships on time?

- No
- Yes

Q9 Will you be taking a research year in 2020-21 and apply in the 2021-22 otolaryngology application cycle? If you are taking or took an extra year for any other reason, please use an "other" option to explain below.

- Yes, I was highly considering a research year even **before** the COVID-19 pandemic (applying in 2021-22)
- Yes, I will now be taking a research year in large part **because of** the COVID-19 pandemic (applying in 2021-22)
- No, I had no plans for a research year and that has not changed (applying in 2020-21)
- No, I was planning on a research year and have decided to apply instead (applying in 2020-21)
- I am already returning from a research year (applying in 2020-21)
- Other (but applying in 2020-21) ____
- Other (but applying in 2021-22) ____

Display This Question:

If Will you be taking a research year in 2020-21 and apply in the 2021-22 otolaryngology application... = Yes, I was highly considering a research year even <u>before</u> the COVID-19 pandemic (applying in 2021-22)

Or Will you be taking a research year in 2020-21 and apply in the 2021-22 otolaryngology application... = Yes, I will now be taking a research year in large part <u>because of</u> the COVID-19 pandemic (applying in 2021-22)

Q10 You indicated that you will be taking a research year.

What are the motivating factors that influenced your decision?

____

____

____

____

____

Display This Question:

If Will you be taking a research year in 2020-21 and apply in the 2021-22 otolaryngology application... = No, I had no plans for a research year and that has not changed (applying in 2020-21)

Or Will you be taking a research year in 2020-21 and apply in the 2021-22 otolaryngology application... = No, I was planning on a research year and have decided to apply instead (applying in 2020-21)

Q11 You indicate that you will not be taking a research year.

What are the motivating factors that influenced your decision?

____

____

____

____

____

End of Block: 2. Applicant Information

Start of Block: 3. Applying this year

Display This Question:

If Will you be taking a research year in 2020-21 and apply in the 2021-22 otolaryngology application... = No, I had no plans for a research year and that has not changed (applying in 2020-21)

Or Will you be taking a research year in 2020-21 and apply in the 2021-22 otolaryngology application... = No, I was planning on a research year and have decided to apply instead (applying in 2020-21)

Or Will you be taking a research year in 2020-21 and apply in the 2021-22 otolaryngology application... = I am already returning from a research year (applying in 2020-21)

Or Will you be taking a research year in 2020-21 and apply in the 2021-22 otolaryngology application... = Other (but applying in 2020-21)

Q12 You indicated that you are currently planning on applying this year.

Given the restrictions on rotations, do you believe you will have an opportunity to complete at least 1 otolaryngology sub-internship for the 2020-21 application cycle?

- ○ Definitely yes
- ○ Likely yes
- ○ Unsure
- ○ Likely not
- ○ Definitely not
- ○ I have already completed a sub-internship

Display This Question:

If Will you be taking a research year in 2020-21 and apply in the 2021-22 otolaryngology application... = No, I had no plans for a research year and that has not changed (applying in 2020-21)

Or Will you be taking a research year in 2020-21 and apply in the 2021-22 otolaryngology application... = No, I was planning on a research year and have decided to apply instead (applying in 2020-21)

Or Will you be taking a research year in 2020-21 and apply in the 2021-22 otolaryngology application... = I am already returning from a research year (applying in 2020-21)

Or Will you be taking a research year in 2020-21 and apply in the 2021-22 otolaryngology application... = Other (but applying in 2020-21)

Q13 Do you believe you will have acquired the clinical exposure and training required to function as an intern in otolaryngology by graduation?

- ◯ Definitely yes
- ◯ Likely yes
- ◯ Unsure
- ◯ Likely not
- ◯ Definitely not

End of Block: 3. Applying this year

Start of Block: 4. Thoughts on the application

Q14 Do you believe the evaluation of candidates in the 2020-21 cycle by otolaryngology residency programs will change as a consequence of the COVID-19 pandemic?

- ◯ Yes
- ◯ Maybe
- ◯ No

Display This Question:

If Do you believe the evaluation of candidates in the 2020-21 cycle by otolaryngology residency prog... = Yes

Or Do you believe the evaluation of candidates in the 2020-21 cycle by otolaryngology residency prog... = Maybe

Q15 How may evaluations of candidates change, in your opinion?

____

Q16 In your opinion, what are usually the three most important factors in judging an otolaryngology candidate? Assume a cycle not impacted by COVID.

- ☐ USMLE Step 1/ COMLEX Level 1 score
- ☐ Letters of recommendation in the specialty
- ☐ Medical Student Performance Evaluation (MSPE)
- ☐ Personal Statement
- ☐ Grades in required clerkships
- ☐ Perceived commitment to the specialty
- ☐ Personal prior knowledge of the applicant
- ☐ Alpha Omega Alpha (AOA) membership
- ☐ Perceived interest in the program
- ☐ Graduate of highly regarded US medical school
- ☐ Demonstrated involvement and interest in research

Q17 In your opinion, what will be the three most important factors in judging an otolaryngology candidate in the 2020-21 cycle impacted by COVID-19?

- ☐ USMLE Step 1/ COMLEX Level 1 score
- ☐ Letters of recommendation in the specialty
- ☐ Medical Student Performance Evaluation (MSPE)
- ☐ Personal Statement
- ☐ Grades in required clerkships
- ☐ Perceived commitment to the specialty
- ☐ Personal prior knowledge of the applicant
- ☐ Alpha Omega Alpha (AOA) membership
- ☐ Perceived interest in the program
- ☐ Graduate of highly regarded US medical school
- ☐ Demonstrated involvement and interest in research

Q18 In your opinion, are there particular types of candidates who are especially disadvantaged in light of the COVID-19 pandemic?

____

End of Block: 4. Thoughts on the application

Start of Block: 5. Virtual Interviews

Display This Question:

If Will you be taking a research year in 2020-21 and apply in the 2021-22 otolaryngology application... = No, I had no plans for a research year and that has not changed (applying in 2020-21)

Or Will you be taking a research year in 2020-21 and apply in the 2021-22 otolaryngology application... = No, I was planning on a research year and have decided to apply instead (applying in 2020-21)

Or Will you be taking a research year in 2020-21 and apply in the 2021-22 otolaryngology application... = I am already returning from a research year (applying in 2020-21)

Or Will you be taking a research year in 2020-21 and apply in the 2021-22 otolaryngology application... = Other (but applying in 2020-21)

Q19 How has the COVID-19 pandemic affected your confidence in matching into otolaryngology for the 2020-21 cycle?

- ○ More confident I will match
- ○ Unchanged
- ○ Less confident I will match

Display This Question:

If Will you be taking a research year in 2020-21 and apply in the 2021-22 otolaryngology application... = No, I had no plans for a research year and that has not changed (applying in 2020-21)

Or Will you be taking a research year in 2020-21 and apply in the 2021-22 otolaryngology application... = No, I was planning on a research year and have decided to apply instead (applying in 2020-21)

Or Will you be taking a research year in 2020-21 and apply in the 2021-22 otolaryngology application... = I am already returning from a research year (applying in 2020-21)

Or Will you be taking a research year in 2020-21 and apply in the 2021-22 otolaryngology application... = Other (but applying in 2020-21)

Q20 Would a move toward video interviews change the number of programs to which you apply?

- ○ Yes, I will likely apply to <u>more</u> programs
- ○ Yes, I will likely apply to <u>fewer</u> programs
- ○ No, I will likely apply to the <u>same</u> number of programs

---

Display This Question:

If Will you be taking a research year in 2020-21 and apply in the 2021-22 otolaryngology application... = No, I had no plans for a research year and that has not changed (applying in 2020-21)

Or Will you be taking a research year in 2020-21 and apply in the 2021-22 otolaryngology application... = No, I was planning on a research year and have decided to apply instead (applying in 2020-21)

Or Will you be taking a research year in 2020-21 and apply in the 2021-22 otolaryngology application... = I am already returning from a research year (applying in 2020-21)

Or Will you be taking a research year in 2020-21 and apply in the 2021-22 otolaryngology application... = Other (but applying in 2020-21)

Q21 Would a move toward video interviews change the number of interviews you choose to attend? How and why?

- ○ Yes, I will likely attend more interviews ____
- ○ Yes, I will likely attend fewer interviews ____
- ○ No, it will likely not affect the number of interviews I attend ____
- ○ Unsure ____

Display This Question:

If Will you be taking a research year in 2020-21 and apply in the 2021-22 otolaryngology application... = No, I had no plans for a research year and that has not changed (applying in 2020-21)

Or Will you be taking a research year in 2020-21 and apply in the 2021-22 otolaryngology application... = No, I was planning on a research year and have decided to apply instead (applying in 2020-21)

Or Will you be taking a research year in 2020-21 and apply in the 2021-22 otolaryngology application... = I am already returning from a research year (applying in 2020-21)

Or Will you be taking a research year in 2020-21 and apply in the 2021-22 otolaryngology application... = Other (but applying in 2020-21)

Q22 In the event of video interviews, do you believe you will gather sufficient information to make an informed decision about your rank list?

- Yes
- Unsure
- No

Display This Question:

If Will you be taking a research year in 2020-21 and apply in the 2021-22 otolaryngology application... = No, I had no plans for a research year and that has not changed (applying in 2020-21)

Or Will you be taking a research year in 2020-21 and apply in the 2021-22 otolaryngology application... = No, I was planning on a research year and have decided to apply instead (applying in 2020-21)

Or Will you be taking a research year in 2020-21 and apply in the 2021-22 otolaryngology application... = I am already returning from a research year (applying in 2020-21)

Or Will you be taking a research year in 2020-21 and apply in the 2021-22 otolaryngology application... = Other (but applying in 2020-21)

Q23 In the event of video interviews, do you believe residency programs will gather sufficient information to make an informed decision about your candidacy?

- Yes
- Unsure
- No

Display This Question:

If Will you be taking a research year in 2020-21 and apply in the 2021-22 otolaryngology application... = No, I had no plans for a research year and that has not changed (applying in 2020-21)

Or Will you be taking a research year in 2020-21 and apply in the 2021-22 otolaryngology application... = No, I was planning on a research year and have decided to apply instead (applying in 2020-21)

Or Will you be taking a research year in 2020-21 and apply in the 2021-22 otolaryngology application... = I am already returning from a research year (applying in 2020-21)

Or Will you be taking a research year in 2020-21 and apply in the 2021-22 otolaryngology application... = Other (but applying in 2020-21)

Q24 What would you like offered during a video interview in order to make an informed decision about an otolaryngology program? Please select all that apply

- [ ] Informal chats with residents
- [ ] Virtual facility tour
- [ ] Opportunity to chat with co-applicants
- [ ] Joining a didactic lecture
- [ ] Program director Q&A/presentation
- [ ] Other ____

End of Block: 5. Virtual Interviews

Start of Block: 6. Final Question

Q25 Are there any outstanding questions or concerns you have about your application you would like to see addressed by otolaryngology program directors? If there are none, you may skip this question.

____

End of Block: 6. Final Question
