# Supplementary material for: Otolaryngology Match 2020-21: Survey of Prospective Applicants in the Setting of COVID-19
Source: Ann Otol Rhinol Laryngol. 2020 Aug 19;130(5):450–8. doi: 10.1177/0003489420952470 (PMC7481654; doi:10.1177/0003489420952470)
Supplement: Supplemental_Figure_2_-_Revised – Supplemental material for Otolaryngology Match 2020-21: Survey of Prospective Applicants in the Setting of COVID-19 [file Supplemental_Figure_2_-_Revised.pdf]

Appendix B. Thematic Groupings for Free Text Responses

Note 1) The order of responses does not correspond between tables
Note 2) We did not exclude the 2 individuals who indicated they are applying next year from this document
Note 3) We have suppressed certain responses in which we felt there were potentially identifying factors with an "X" and attempted to describe what was suppressed within brackets. For instance, if an applicant stated "Los Angeles," we would substitute "XXX [city]."

Appendix B-1) Thematic groupings for an "other (MD/PhD, joint degree, etc.)" in response to "What was your original graduation year, upon matriculation?"

Response	Thematic Grouping		
	MD/PHD	Reapplicant	Multiple Research Years
research years			1
Started in 20XX, X year PhD, plan to graduate in 2021	1		
MD/PhD	1		
Reapplicant. Graduated class of 20XX		1	
20XX, did not match and now pursuing a joint degree program		1	

Appendix B-2) Thematic groupings for an “other” in response to “How has your time been spent during the COVID-19 pandemic?”

Response	Thematic Groupings	
	Virtual Classes	Recoded into Existing Variable
Online med school class	1	
Online courses through medical school	1	
Family responsibilities (providing childcare)		Respondent has already clicked off "family time"
Online courses	1	
Virtual clerkship	1	
adopted a XXX [pet]		Respondent has already clicked off "family time" & "Hobbies/Personal Development"
Non clinical coursework	1	
Preparation for Residency		Recoded into "non-USMLE studying"
Online "Clerkships"	1	

Appendix B-2) Thematic groupings for an "Other (but applying in 2020-21)" in response to "Will you be taking a research year in 2020-21 and apply in the 2021-22 otolaryngology application cycle?"

Response	Thematic Groupings		
	Reapplicant	Considering a research year	Recoded into Existing Variable
reapplicant	1		
preliminary XXX year after failing to match this year	1		
I had not been planning on taking a research year, but I am now considering it as an alternative to applying in 2020-2021.		1	
Taking a research year and applying this year			As this individual indicated they were in the original class of 2020, this implies that they are finishing their research year, and so they were recoded into "already returning from a research year"
I have considered taking a research year in large part because of COVID, but do not plan to do so at this time.		1	
Both of my research year opportunities are on hold or cancelled because of research funding and new COVID-19 restrictions			As this individual had written that they would be applying this year, we reclassified them as "No, I was planning on a research year and have decided to apply instead (applying in 2020-21)"
I did not match originally and will be re-applying this year after planning a year for research and joint degree--unrelated to COVID.	1		

Appendix B-3) Thematic groupings for an "You indicated that you will not be taking a research year. What are the motivating factors that influenced your decision?"

Did the individual ever plan to take a research year and change their mind?	Response	Thematic Groupings						
		Sufficient research already conducted	External factors influencing match timing (couples match, military commitments)	Research not aligned with career goals	Financial considerations	Desire to avoid delaying residency	Insufficient opportunities for research	Uncertainty regarding the 2021-22 match
No	My faculty advisor told me that I did not need a research year to have a competitive application	1						
No	I had an interest in ENT early on and was able to participate in research throughout my time in medical school.	1						
No	I feel as I have enough research on my application that I do not require a research year.	1						
No	Decent research background and productivity during medical school	1						
No	Applied for XXX [research fellowship] and was rejected.						1	
No	I believe I have a decent amount of research on my application and that I am a competitive applicant.	1						
Yes	I thought long and hard about taking a research year but I ultimately do not think that taking a research year will make me a stronger applicant. With my current, though limited, clinical experience and research I think I have solidified my interest and aptitude in Otolaryngology. Additionally, I will be participating in the couples match and I do not think that taking a research year would be in my and my XXX [significant other's] best interest at this time.		1					
Yes	I like having a structured schedule and when the pandemic began it was similar to a research year structure where I would just work on research all day, and I didn't like it.			1				
Yes	Perceived competitiveness of the following cycle, inability to adequately relocate to place of research year, too much uncertainty in general of any decision							1
No	I have been interested in ENT since I was a first year and have been involved since then, so I have some letters secured	1						
No	I am not passionate about research and I'd rather just be done with medical school already			1		1		
Yes	Additional time with delay of ERAS	1 because it implies that they have additional time to do research						
No	Not wanting to delay career					1		
No	I feel that I am ready to move forward with my current application as it stands.							
No	Finances, Previous Research Experience, No clear benefit of applying next year vs this year.	1			1			1
No	Grades/step 1 score, enough research, don't want to wait a year, and likely have an option for research year if match unsuccessful	1				1		
No	I already had the opportunity to participate in ENT research during medical school and felt like I was prepared to enter residency without taking more time off.	1				1		
No	I did not want to delay my training and felt that I would be competitive without one. I enjoy research but not enough to dedicate a full year to it.					1		
No	Cost, personal applicant profile metrics, time				1			
No	N/A							
No	I believe that I already have enough research where an extra year would not be beneficial to my application, and is not amenable to my future plans.	1				1		
No	Home institution has strong research program that I got involved with M1 year and I feel like research is one of the strongest parts of my application. Additionally, I took X years off after undergrad prior to medical school so no more gap years for me	1				1		
No	Don't have a good option under short timing for doing a productive research year						1	
No	Desire to start ENT residency as soon as possible.					1		
No	Sufficient pre-medical school research experience.	1						

No	I want to be an otolaryngologist who conducts research, not a researcher who is also an otolaryngologist. I believe I can match without "needing" a research year.			1				
No	I can not financially afford to take a research year.				1			
No	I would like to get back to clinical rotations ASAP and do not want to delay residency any longer.					1		
No	Had the opportunity to work on several research projects in med school, didn't want to lengthen timeline.	1				1		
No	I was not interested in taking a research year before and have hopefully obtained an adequate research CV for my application. I am also couples matching and have a home program.	1	1					
No	I don't think a research year will affect my outcome in the match too greatly compared to next year. Also no guarantee conditions are better regarding away rotations, etc next year							1
No	Adequate research experience without year	1						
No	I have already done a XXX [graduate degree]	1						
No	I had been on the fence about taking a research year before the pandemic started. I ultimately decided not to, because I felt that I had enough research projects either completed or in progress. Personally, I felt that I would not be able to stay motivated/productive during an unstructured research year, which is what was available to me at my home institution. I briefly revisited this decision in March when the pandemic started, and after discussion with my advisors, ultimately came to the same conclusion, with the understanding that any differences or disadvantages that would occur during this cycle would be uniform.	1						
No	XXX [External obligation], don't have the option to take a research year		1					
No	More competitive next year, All applicants equally affected this year							1
No	Less competition this year, already have research on resume	1						1
Yes	Cost				1			
No	I have what I hope is already a sufficient amount of research on my CV to be a competitive applicant. Furthermore, I have a XXX [Significant Other] and XXX [number] kids, and I don't have the financial means to take another year off while loans accrue interest.	1	1		1			
No	I have done research throughout medical school and I did not feel the need to take a research year.	1						
No	Desire to begin residency, potential negative views of taking a research year					1		
No	lots of research productivity during this time; feel like COVID levels the playing field a little bit and gives me a better chance	1						
No	I have strong research background and don't see any reason to do so	1						
No	I feel that participating in a year of research does not align with my future goals and research is not something that I am particularly interested in.			1				
No	I took several years off before starting medical school and didn’t want to delay any further. I was also fortunate to know I wanted to do ENT from the beginning and have been doing research for a little while now. I didn’t feel like a research year would add much to my application.	1				1		
No	I already have conducted a fair amount of research and felt that an additional year off would not be absolutely essential to match into otolaryngology for this upcoming cycle	1						
No	wanting to move on with my life.					1		
No	People from my school (XXX [US region]) typically don't take research years when applying ENT. I have good scores, grades, published research (outside of ENT), and will ENT research in progress by the time we apply, so I feel like I'll be as strong as I can be by the time we apply.	1						
No	I've conducted research in medical school and did not feel the need to take a full year for research.	1						
No	I am working on research now	1						
No	Already have plenty of research experience	1						
No	I have several publications and do not believe that an extra year of research will significantly improve my application. Additionally, there is no guarantee that the next application cycle will return to complete normalcy, and I would rather compete with students in the exact same position as I am rather than allowing the situation to differentiate.	1						1

No	Financial difficulties; prior and ongoing exposure to ENT research	1			1			
Yes	Both of my research year opportunities are on hold or cancelled because of research funding and new COVID-19 restrictions						1	
No	Feel ready to apply							
No	I already have a substantial amount of research experience and was able to join X [number] additional projects during the past two months in which I was not allowed in the hospital. Ultimately, the only detriment to my application as a direct result of the COVID-19 pandemic was that I was unable to perform away rotations. However, since this is a universal policy that affects practically all applicants, I did not feel this was significant enough to warrant taking an extra year off from my desired career path.	1						
No	I have completed a few projects during medical school, and I don't feel that a research year would add significantly to my application.	1						
No	I have enough research in my CV already, and research is not necessarily a focus of my career goals.	1		1				
No	N/a							
No	I have thoroughly explored career options and do not feel that I need further validation in the form of research to confirm my career choice. I have completed enough research during medical school and have other factors to feel confident that I have a reasonable chance of matching.	1						
No	I am currently involved in X [number] research projects, one that I expect to be submitted before applications are due, the other maybe later this year. My other stats are generally good.	1						
No	Worked on research diligently throughout my early time in medical school	1						
No	I have already done a lot of research	1						
No	The deficits in my applications are scores and not research; thus, a research year would not improve the quality of my application.							

Appendix B-4) Thematic groupings for an “How may evaluations of candidates change, in your opinion?”

Response	Thematic Groupings					
	Known candidates favored preferentially	Decreased emphasis on letters	Greater emphasis on Scores and/or Research	Greater emphasis on clerkship grades or MSPE	Greater emphasis on medical school reputation	Greater emphasis on holistic evaluation with accommodations for gaps in application
Programs will not get to know me as a person because there are no away rotations and interview will be remote.	1					
I think the final rank process that was largely based on in person interview will definitely be changed. The virtual interview might be used is adjunct to social media presence and other factors of the application ie LoR, comments from required clerkships, might be weighed more heavily.						
Larger emphasis on Step 1 scores; no away rotations can be considered because they are unavailable this year			1			
More focus on CV			1			
Virtual interviews will work negatively against non-home applicants.	1					
Candidates will be judged more on their applications than their personalities.			1			
I imagine that clinical grades in key courses/electives, standardized tests, and extra-curricular involvement (volunteering, research) will be even more strongly valued. While these factors are always valued highly in Otolaryngology applications, I think PDs may look elective experience and research that suggests that a student would perform well as an Otolaryngology resident. I think programs will also closely evaluate how students spent their time during this pandemic.			1	1		
more superficial and less focused on clinical abilities.			1			
remote/virtual interviews, favoring home candidates	1					
Schools with well-established, prominent home departments will exceedingly prepare their candidates better for the match. The resources (i.e. research opportunities) are also going to be much more abundant at such places. Students without such things are at a great disadvantage.					1	
Focused more on the numbers (scores) and letters as well as medschool rank/reputation.			1		1	
Spend more time getting to know students since away rotations are no longer going to be widely offered. Programs will likely be interviewing more candidates, which makes for a more competitive year.	1					
It will be more difficult to evaluate candidates in virtual format.	1					
Greater preference to take home students who have rotated in the department compared to unknown students from other institutions	1					
not sure						
I personally believe I do pretty well in person and was looking forward to going on away rotations as well as interviewing in person versus virtually.	1					
More likely to accept students from own institution	1					
I feel that candidates with home residency programs will have an advantage over those without home residency programs due to the fact that they will be able to complete electives in Otolaryngology at their home institution.	1				1	
More emphasis on test scores and other "metrics" without away rotations, more emphasis on school prestige			1		1	
Virtual interviews will limit how your personality comes across and unable to interact with residents to see if you fit. Will restrict many students hoping to impress on away rotations.	1					
I think that there may be more reliance on research and test scores in this application cycle. I worry that it will be difficult to indicate regional preference without away rotations.			1			
Harder to impress without Aways.	1					
Programs will keep home applicants	1					
I think more weight will be given to students from their institution. among other possible changes. including, less weight on clerkship grades as less students have been able to complete all core clerkships.	1					
Holistic approach rather than traditional metrics, greater emphasis on personal characteristics obtained from letters of recommendation/personal statement/etc						1
Institutions are more likely to seek candidates who they are familiar with, and those with home otolaryngology programs will therefore have a particular advantage.	1					

Greater emphasis on reputation of home program/medical school and personal connections. More emphasis on Step 1 scores. Less emphasis on other personality traits or interpersonal or operative skills, since these cannot be observed at aways.	1		1		1	
Step scores, grades, research, and what medical school applicants go to will likely carry more weight. Letters of recommendation will carry less weight since all will be from home program.		1	1		1	
Letters of recommendation from home program with hold more weight without away rotations. Programs may not get to know candidates as well through virtual interviews, so may rank home applicants more highly than usual	1					
I think it will be more based on how an applicant looks on paper.			1			
More preference for home students	1					
XXX [Removed due to identifying information]						
Programs wont be able to see candidates "in action" during away rotations	1					
Less emphasis on extracurriculars.						1
Personal connections, either through familiar letter writers or calls on behalf of the student, will be of increased importance when students have limited contact with outside institutions.				1		
less emphasis on away rotations		1				
Cannot evaluate candidates from away rotations, may match more home students, fewer LOR	1	1				
My hope is that programs will be understanding of lost opportunities due to COVID 19.						
Unsure. But I could imagine that getting LORs in the specialty is going to be more difficult for students now, given limited time to rotate, no aways, etc.		1				
Without in-person interviews and sub-internships, I think programs will have to evaluate applicants much more based on their "on-paper" application. There will be less of a chance to evaluate how participants function as part of a clinical team and less of a chance just to get to know them as people.	1		1			
I feel that emphasis may be shifted even more towards step 1 scores, grades, and research given that they are objective measures and the other highly important subjective measures (aways, rec letters) are disproportionately impacted/restricted by the pandemic			1			
I think it will be very difficult to evaluate students in this new clinical setting, lack of research fairs and poster opportunities, without away rotations, and with virtual interviews. I am hopeful that this will not negatively affect us but am just honest that it will be very different than the past years.	1					
Less emphasis on amount of letters since that has been affected by the away rotation status		1				
I think convenience metrics will play a greater role (USMLE Step scores, clerkship grades, pedigree)			1		1	
Perhaps objective measures will become even more important if interviews are not conducted in person. Also programs may be more inclined to rank their home institution applicants higher.	1		1			
I think more emphasis will be placed on test scores and publications. Recommendation letters will be tricky as not everyone has a home program...		1	1			
Step 2 scores should be weighed less, LORs are difficult to come by		1				
School rank and home program will become much more important factors. Students without home programs will be strongly disadvantaged. Obtaining credible letters for students without home institutions will be extremely difficult.	1				1	
More focus on USMLE, academic CV, research productivity instead of on-site away elective			1			
More holistic approach						1
Benefit of candidates with big names and prominent home institutions					1	

programs will have to be more willing to interview/ take chances on candidates that they have never met since there will not be externships						
More stringent screening cutoffs, including Step 1 and research publications			1			
I believe that who you know and what school you went to will become more important now that students do not have the opportunity to showcase their skills or their personalities during away rotations. I worry that now candidates will be evaluated in a much less holistic, more calculated manner.					1	
with virtual interviews and no away rotations, programs will not be able to meet candidates in person.	1					
It may lean more heavily on scores since there won’t be always to factor. Interviews may also be less thorough when remote.			1			
More interviews offered, more preference to home applicants, more weight placed on AI LOR	1					
less dependence of Step 2; less in-person impressions, etc	1					
will be even more inclined toward scores and transcript/cv			1			
Without away rotations or sub-Is, there is no way to prove my skills and knowledge translate to the field of ENT because I do not have a home program.	1					
It’ll be driven largely by letters of recommendations, personal contacts, and board scores (i.e. likely less holistic than in usual years)			1		1	
Depending on geographical location and school, students may likely have difficulty completing sub-internships in otolaryngology and subsequent letters of recommendation. For students without a home department, which I am one, I would hope for this cycle, PDs are more open to accepting letters of recommendation from non-academic otolaryngologists						
not sure						
more reliance on 3rd year performance, potentially.				1		
For one, there are no away rotations to audition and showcase your clinical abilities. Programs will no longer be able to rely on getting to know an applicant through away rotations. Additionally, virtual interviews will provide its own obstacles. Getting to know someone over Zoom will be a challenge vs meeting someone in person. It is difficult to establish cadence, rapport and read body language over virtual interactions.	1					
More emphasis may be placed on the reputation of an applicant's medical school and/or personal prior knowledge of an applicant obtained through a home sub internship rotation.	1		1		1	
May be based more on what they see on paper			1			
Interviews will be harder to assess applicants virtually	1					
I do think home institutions will rank their own students higher. I hope this does not end up being true, but I think it is likely.	1					
Step scores will become more important, which is disappointing.			1			
applying a more holistic approach, recognizing that not everyone will have access to well-known faculty recommendations. Understanding that some students' schools have decided to change clerkship grades to pass/fail and to not hold that against students (eg, those who now cannot "honor" IM/Surgery).						1
They will only be from one institution and not from physicians at away rotation locations where we would like to match.	1					
More flexibility with app requirements						1
I would assume there is an increased emphasis on other factors of one's application, such as letters of recommendations, board scores, grades, and research experience. The shift towards virtual interviews will allow for increased access to applicants with limited financial resources to have otherwise scheduled flights/accommodations for interviews at a moment's notice. However, it will be an additional challenge for most in that the format is different than that to which we have accustomed ourselves.			1			
The specialty will be more dependent on non-OTO letters and evaluations. The letters from OTO evaluators may not be able to comment on the clinical performance (OR or clinic). of the applicant.						1
Evaluations will become more lenient of applicants as a whole, and more weight will be put on examination test scores. For a positive, maybe evaluators will put less weight in the identity of letter writers as many students will be unable to complete aways and will be more interested in the wholistic approach rather than "connections."		1	1			1
More of a preference toward universities with a strong Otolaryngology program, more of an emphasis on research and publications where letters of recommendation may be lacking		1	1		1	
Greater emphasis on home students and scores/comparable metrics	1		1			

XXX [comment some ENT sub-I’s are now obligatory P/F]. Grades probably thus matter less, and letters of recommendation matter more. Furthermore, Step CK probably doesn't matter because so many students won't be able to take the exam prior to application season.						
Less emphasis on step2. More reliance on LoR from home programs						
Away rotation letters provide important info on potential fit of applicants that is less biased than from the home program. I feel like many students are going to appear quite similar on paper and other factors like Step 1 score and school reputation will be more important.		1	1			
Programs may put more emphasis on research and other experiences rather than certain grades due to disruption in CK and clerkship experiences. Also, aways are not going to happen, which were previously pivotal in the application			1			
There will likely be higher bias towards those who have mentors who are well known. Phone calls will likely become more important than they have been in prior cycles, and students from higher ranked schools will likely perform better in this cycle					1	
less holistic, more focused on numbers like Step 1 & GPA			1			
Applicants that have home programs with renown ENT faculty that are able to acquire letters will have more impressive applications. T20 med schools also have reputations that are valuable when programs are recruiting applicants. Applicants with small or non-existant home programs may not receive an opportunity to acquire letters or experience needed to match.					1	

Appendix B-5) Thematic groupings for an "In your opinion, are there particular types of candidates who are especially disadvantaged in light of the COVID-19 pandemic?"

Response	Thematic Groupings				
	Students with no home program or limited mentorship opportunities	Graduate of an osteopathic (DO) institution	Candidates relying on Step 2CK or away rotations to bolster their application or make up for a perceived weakness	Candidates with a particular regional or institutional preference	Candidates with a late interest in ENT
No					
I think candidates without home programs or without the opportunity to rotate in an Otolaryngology sub-I. Also, students relying on a good Step 2CK to help a below average Step 1 score might be at a disadvantage due to difficulty securing a test date, uncertainty of test cancellation during dedicated study period and overall stress about the COVID pandemic and loved ones during a very critical period of time where performance on a single test day could contribute to not being able to pursue the specialty the applicant is interested in.	1		1		
People without home programs; people who wanted to do aways in order to go somewhere specific; people who were counting on Step 2CK to make up for a bad Step 1 score	1		1	1	
Borderline candidates, those without home program	1		1		
Students without home programs and students who are weaker on paper (board scores or research).	1		1		
DO students, students without an ENT program at their home institution, students with marginal Step 1 scores whose step 2 CK was affected	1	1	1		
Students without a home Otolaryngology program, students who choose to pursue Otolaryngolgoy later in their medical school careers	1				1
yes, students with poor scores, from not as competitive schools, etc			1		
those without home programs or hoping to match at an institution they were looking to do an away at, those with unstable WiFi/Internet, those who have not completed a sub-internship at their home program	1			1	
Students without strong home programs and connections to advocate on ones behalf when away rotations students could "self-advocate" by having outstanding clinical performance. Small or non-existent home program students	1				
Students of schools without a program, makes securing letters from strong prominent ENTs more difficult. Was hopeful and aiming to do 3 aways to obtain letters and showcase myself.	1				
Those who attend schools without ENT programs, those who were relying on away rotations to bolster LORs, those who are still undecided	1				1
Students from schools without strong home departments	1				
Students without home otolaryngology programs, students from small lesser-known home departments	1				
people without programs	1				
Those without home ENT programs, those who are not as strong on paper, yet may be better in person	1		1		
Students from programs with no or average ENT programs (vs. powerhouse institutions)	1				
Those without home residency programs, those coming from smaller medical schools	1				
Students from schools without home programs, students who were hoping to use strong Sub-I performances to overcome prior weaknesses, DO students, students with low step 1 scores, students from non-top tier schools	1	1	1		
DO students and students with below-average Step 1 scores/grades		1	1		
I believe that this cycle will disadvantage students without a home program, students who were hoping to use away rotations to bolster other portions of their application and students without enough elective time to explore ENT.	1		1		1
Participants without home programs will likely be disadvantaged.	1				
Students without home	1				
Those who do not have a home program or a mentor in the field of ENT to guide them through this process. Those who do not have a wealth of research experience and were banking on away rotations to shine and get letters	1		1		
candidates without home institutions, candidates from non-Top 40 medical schools, candidates from medical schools with small/less academic otolaryngology programs	1				
Those without home OHNS program, those without high STEP scores or research output	1		1		
Underrepresented minorities, who already receive disproportionately lower clerkship grades due to structural racism. Also given the research showing a racial disparity in AOA membership (not only for black students, but also for Asian students). Also students without home programs or strong support from their home programs.	1				

Those without a home OHNS program.	1				
Applicants who did not get to complete a core surgery clerkship because their 3rd year was cut short					
Those with no home rotation, and those who decided relatively late on ENT.	1				1
Students without home programs and were relying on away rotations	1				
Reapplicants who decided to pursue a preliminary surgery year and those with average USMLE scores without programs	1				
Those from schools without home programs and less "prestigious" faculty to write them letters of rec from home instititiuon	1				
Students without home programs.	1				
Students without a home program or easy access to ENT faculty	1				
no home program	1				
Those who come from medical schools without well-known faculty or home otolaryngology department	1				
Those without home programs.	1				
Those of us who became interested in oto late and who were already scrambling to try to get research opportunities lined up. These opportunities have been delayed significantly during cover, and I can't imagine getting any meaningful research done prior to application.					1
Applicants who have good grades/scores/CV but are not people who teams would enjoy working with					
candidates from smaller schools, or those without a home program	1				
Candidates that were relying on away rotations and another opportunity for success on a USMLE exam will likely be disadvantaged. Students at lower tier medical schools will also be disadvantaged. It will be difficult to shine in a virtual setting.			1		
DO students and those w/o a home program	1	1			
Those without away programs, those who are unable to take Step 1 or Step 2 CK (for example, to 'compensate' for a poor Step 1)	1		1		
Those without home otolaryngology programs or adequate mentoring.	1				
Absolutely: applicants from less well-recognized med schools, applicants without otolaryngology at their home institution	1				
N/a					
those without home institutions, those from mid tier medical schools	1				
Students without home institutions. Students without home institutions and no strong connection to a program. Students lacking mentorship from an academic ENT program-affiliated mentor. Students without away rotation letters.	1				
IMG, DO, Low-to-mid tier MD students; Those without home programs; Those without connections	1	1			
Candidates without a home institution	1				
No home institutions, those who get sick with covid	1				
those without a home program	1				
Students without a home institution or students from a mid tier US MD School and below	1				
Candidates without home programs are disproportionately disadvantaged by this situation. They have the distinct disadvantage of not having a home program that can not only give you a Sub-I when no one else will, but will also advocate for you and write letters for you with names that open doors.	1				
those without home ENT programs	1				
Those who don’t have an ENT program at their school. Those who would like to move geographical regions.	1			1	
I think the playing field is relatively the same but the cycle will be handled differently					
people depending on aways			1		
Those who don't have home programs, come from no well-known universities, and those who performed poorer on step exams	1		1		
Students without home programs. Students who decided they are interested in ENT late in their schooling. Students that don't have connections to ENT programs and/or faculty.	1				1
Candidates without home programs, IMG/FMG, candidates hoping to leave their home institution candidates from less well-known medical schools	1			1	
Those without a home program or those who come from a lesser known school as this makes letters of recommendations difficult outside of a non-academic otolaryngologist	1				
people without much clinical exposure					
those with no home ENT program.	1				
Possibly students without a home institution. However, they are able to complete aways which in a way is a bonus to be able to audition while other students are unable to do so.	1				
As an applicant from a medical school without a home otolaryngology program, I feel disadvantaged in light of the likely cancelation of most away rotations. I feel I may be unable to obtain expected letters of recommendation and otherwise demonstrate my skills to potential residency programs.	1				
Students without a home program, students with lower stats	1		1		
Those without a home residency program	1				

those who don't have home programs and those who are more impacted by COVID-19 in terms of disruption to core clinical rotations/graduation requirements. also, a ton of research that I was doing on my research year was lost because of the sudden disruption.	1				
Candidates at non-top 50 research schools are potentially disadvantaged, but those without home ENT programs are without argument disadvantaged	1				
Couples match applicants, people with lower step scores but high grades, people without research, people who haven't had surgery/ENT rotations because they were cancelled due to COVID					1
Those from programs who do not have well-known letter writers					
Students without a home Otolaryngology program. Students that are great sub-is (off paper)	1		1		
Yes					
I believe candidates who have no home otolaryngology departments and were relying on away rotations to demonstrate their talent and acquire letters of recommendation are undoubtedly the most negatively impacted. Individuals with lower tier Step 1 scores and those who attend medical schools that are not highly ranked will likely be impacted as well. However, with the increased ease in accepting virtual interviews, programs may find their interview pool shift significantly even with a deliberate focus on factors other than away rotation performance.	1		1		
Yes, applicants from schools without a home program. With the new guidance on away rotations, it may be very difficult for these applicants to get an away rotation.	1				
Those without a home program, and those without as much money.	1				
Yes - it seems that students from universities without a strong Otolaryngology program may have more challenges.					
Students at non Top 40 NIH Medical Schools, students without a home program, reapplicants	1				
Those with weak Step1 scores are likely highly impacted negatively. Those without home rotations are also highly impacted negatively.	1		1		
I believe this cycle will be heavily regionally biased. I'm that sense, one could be disadvantaged if one was hoping to match at a program across the nation, for example.				1	
Those without a home program. Those that are not from highly ranked medical schools. Those who decided to pursue otolaryngology later during medical school.	1				1
DO, no home program, small/mid-tier programs, low-research programs, low-connections in the field.	1	1			
Students from schools that are not well known					
people who have good communication skills and interact well with faculty (away rotations)					
DO applicants, applicants with no home programs, applicants from lesser-well known programs, IMGs, applicants who are unable to sit for USMLE Step 1 and/or complete required clerkships by ERAS	1	1			

Appendix B-6) Thematic groupings for reasons behind "Yes, I will likely attend <u>more</u> interviews" in response to "Would a move toward video interviews change the number of interviews you choose to attend?"

Response	Thematic Groupings			
	Ease of attendance (i.e. less travel)	Increased uncertainty about relative competitiveness	Lessened financial burden	Feel the need to attend more interviews to gauge relative program merits
I think if I received 20 interviews it would be very difficult to attend all of them, however, if they were all virtual then I believe I could possibly do all of them.	1			
I think that the uncertainty around the pandemic will lead me to attend more interviews to ensure that I match.		1		
Less travel cost and time	1		1	
Less time spent on travel	1			
I will not have to consider flight costs, travel plans, or interview dates that are too close together	1		1	
I think it will be hard for me and programs to mutually evaluate/rank one another via a video platform because it is so new. Because of the novelty, I think I will attend more interviews to gather as much information as possible.				1
why not?				
will not need to pay for flights and hotels			1	
I have more chances to obtain interviews and be ranked at more programs. I also need more choices to look at myself because I will not have the in-person experiences otherwise.		1		1
Cost and time are not as much of a factor, therefore I will have availability for more.	1		1	
It will be more feasible and less costly	1		1	
Easier to schedule back-to-back interviews without having to factor in travel time	1			
because it will be easier to attend all of them	1			
Video interviews are more accessible.	1			
Less likely to make a good impression over video conferencing as opposed to in-person away rotations + interviews. Also, peer pressure effect from other applicants also applying/interviewing more.		1		
I will likely attend more due to the decrease in travel expense and time commitment to travel to various institutions.	1		1	
Without enforceable caps, although it would not be good for the overall applicant pool, individual applicants are incentivized to attend the maximal number of interviews that can to increase matching odds. It is a prisoner's dilemma.		1		
No cost of travel and board			1	
I imagine that in the typical application cycle, students are limited in the number of interviews they can accept due to the condensed nature of the interview season. This may be removed by virtual interviews.	1			
Decreased cost of travel and less time to get between locations.	1		1	
Lower cost (both financial and emotional/physical). Greater opportunity to explore programs to find a suitable fit. But it's not driven just by the move towards video interviews-- it's also because I'm scared I won't match because things will be so crazy this application cycle. So I probably would have gone on more regardless of video or not, but with video, I'll probably go on a few more on top of that.		1	1	1
Will cost less and time commitment will be less.	1		1	
Video interviews save a lot of time and money, making it feasible to attend more interview. Without aways, programs will likely rank home applicants higher, so it may require more ranks to match this year.	1	1	1	
It seems more feasible that I would be able to attend more interviews if travel is not a time-limiter.	1			
No cost, no time constraints	1		1	
yes, its more economically feasible			1	
I am worried matching will be more difficult for considering I wont have letters from away programs and thus doing more interviews is to my advantage and less financial cost		1	1	
Less expenses for travel.			1	
The limiting factor will be the dates which interviews are scheduled. Absent an external limit on the number of applications or interviews attended, students will attend as many interviews as possible.	1	1		
Because candidates will not travel for interviews, each one can attend a greater number.	1			
Finances will not be a limiting factor and a it will be harder to interact with programs virtually, necessitating more interviews in order to find the right program.			1	1
Less cost-prohibitive.			1	
Uncertainty about matching, and no financial constraint (travel, lodging, etc)		1	1	
If I don't have as many time constraints since I'm not traveling, there would be no reason to cancel interviews unless they directly conflicted; I'm also saving $ from not traveling	1		1	
Less time to commit per program allowing for more interviews to conduct	1			
Easier to attend interviews if virtual.	1			
I can afford infinite virtual interviews; travel is very expensive and more time consuming	1		1	
less money/logistical challenges; more opportunities to put my face to my name		1	1	
It will be feasible to attend more interviews.	1			
No travel and less time cost	1		1	
Saves money and time	1		1	
It would be feasible to attend more interviews without the hassle of travel. Since number of interviews is so important in matching, having a greater number of interviews would not hurt.	1	1		

With many interviews running on the same day it would be impossible to attend every interview, however without having to fly to different states in back to back interviews, its possible to attend more interviews.	1			
I have no home program, so I will try to attend as many interviews as I receive so that I can maximize my chances of matching.		1		
Less costly and less scheduling overlap	1		1	
It’s easier to schedule and attend so I’ll likely go to all of the interviews I’m eligible for.	1			
Ease of access to interviews, increased chances of finding a good program Fit	1			1
no cost anymore; why not			1	
It is easier to attend interviews from home and also other applicants will do the same	1	1		
Travel will not be a limiting factor.	1			
Definitely! Lower cost, fewer barriers, increase my chances. Certainly won’t go overboard, but will likely take more interviews than I would’ve normally.	1		1	
Without the time and expense of travel, I would likely attend every single interview extended if the dates were possible	1		1	
No traveling cost.			1	
With the cost of travel eliminated, there will be no financial burden in attending more interviews.			1	
Na				
finances and logistic	1		1	
Simply because of the lack of travel time, logistically it should be possible to attend more interviews. Additionally, I would attend as many interviews as possible to expand my rank list to avoid not matching.	1	1		
Won't have to account for travel days. Might have less interviews though because there will be less cancellations though	1			
There is limited financial or emotional cost associated with attending a virtual interview as compared to in-person interviews.			1	
Less travel expenses.			1	
I am concerned that applicants will keep more of their interviews without the restriction of geographical travel, and the guideline of trying to attend about 14 interviews in order to feel safe for the match will no longer hold true. I also hope that programs will be transparent about the number of interviews they are conducting (i.e. whether or not they conduct more interviews) because interviewing more applicants will also make it harder for each individual applicant to judge their level of security in the match.		1		
Since it's physically possible, I'll attend more interviews if available. The limiting factor in the past was just time/physical constraints. This also depends on the number of interview invites I get at all.	1			
It will be more financially reasonable to attend more interviews and if they are offered I will likely accept them (assuming I am still interested in the program).			1	
The lack of financial and time constraint will result in availability to attend more interviews. Also, now that we can't physically be at the institution, I want to experience as many as I can to try to make a rational decision where to rank highest with whatever information I can access.	1		1	1
lower cost			1	
I will be more uncertain about the process		1		

Appendix B-7) Thematic groupings for reasons behind “No, it will likely not affect the number of interviews I attend” in response to “Would a move toward video interviews change the number of interviews you choose to attend?”

Response	Thematic Grouping
	Previously planned on applying and interviewing broadly
Should be interviewing same number of applicants per spot	
I would've planned to attend as many interview as I received. This would not change with Covid. However, because I will be applying to more programs, I will hopefully get more interviews.	1
I was likely to apply to ~80+ programs regardless of COVD-19	1
I was going to apply broadly regardless	1
I have been hopeful that I will be able to attain the sweet spot of 15-18 interview offers and be able to attend them.	
I will try my best to attend every interview.	1
I will probably attend just as many	
I had plans to apply fairly broadly and will likely do so.	1
My application will be the same and I was advised by my program to apply for the same number of programs as before COVID	
I would go to every interview that I receive, either way.	1
No, I will likely attend the same number. I anticipating attending all interviews offered whenever possible.	
I do not have stellar board scores; thus, I will apply to every program irregardless.	1

Appendix B-8) Reasons behind “unsure” in response to “Would a move toward video interviews change the number of interviews you choose to attend?”

Response
I don't know yet. I will definitely be saving money though.
I will still only be able to attend one interview per day, but any scheduling restrictions due to traveling will be removed. All in all, I don't think the amount will be changed much but it'll just be much cheaper.
Would still depend on how many interview invitations I receive.
i would need to know more, i suppose
Hopefully this makes scheduling easier/more flexible. But that's TBD
Hard to tell without knowing format of video interviews
I don't think I would attend more or less interviews than before.

Appendix B-9) Thematic groupings for an "Other" in response to "What would you like offered during a video interview in order to make an informed decision about an otolaryngology program?"

Response	Thematic Groupings		
	Information about local culture and lifestyle	Information about institutional culture among staff and trainees	Research Talks
Information regarding the geographic location and what it is like to live there (difficult to assess without visiting)	1		
Some way to assess resident interactions with attendings - are they friendly? Does there seem to be a rigid hierarchy? etc.		1	
It would be incredibly valuable to have an anonymous Q&A session with the PD and with the residents. There are some sensitive questions that applicants have always been afraid to ask-- like parental leave policies, mental health support, residents not completing the training, etc. This is a great opportunity to increase transparency about these very important topics in a way that protects applicants!		1	
Perhaps it may be useful to pair up with residents for one on one chats or breakout rooms instead of many people in one zoom call.			
Conversation with faculty working in research areas of mutual interest			1
Although I wish to have opportunities to chat with residents and co-applicants, most avaible video services are built such that only one person can talk at any given time. The social etiquette of not "hijacking" the entire conversation strikes a stark contrast to the informal one-on-one conversations that one could still easily have in an in-person, large group setting. I can't imagine getting a sufficient feel for someone's personality purely based on how they interact online.			
Some kind of structured game or activity (i.e. pictionary, themed jeopardy) in a virtual setting might be fun to break the ice on an informal get together.			
I'd like information on living in the area (e.g. expected living expenses, parking, fun things to do in the area, weather).	1		

Appendix B-10) Thematic groupings for an "Are there any outstanding questions or concerns you have about your application you would like to see addressed by otolaryngology program directors?"

Response	Thematic Groupings		
	Program guidance regarding approach to evaluating applicants	Program guidance regarding approach to interview invitations and structure of interviews	Expressing interest in a specific program
How will PDs gather sufficient information about applicants from the virtual interview? Will there be a change in interview programming?	1		
I believe Step 1 scores, clinical ENT experience, and LOR should be looked at with more leniency. I am also worried that programs will expect students to have done significant research during this pandemic.			
Are program directors going to limit the number of residency interview invites?		1	
Will applicants from other institutions be given equitable consideration by programs compared to home students?	1		
Capping applications at a reasonable number (e.g. 30 programs) would prevent interview hoarding, and simultaneously allow PDs to know that all the applications they receive are from authentically interested students, not just "shotgunned" applications from nervous applicants who prefer other programs/regions.		1	
Is there any move towards standardizing when interview invites are sent (like plastics has done), and ensuring that the number of invites sent doesn't exceed the number of spots? It's a bit ridiculous to spend weeks being afraid of leaving our phones, just to make sure we don't reply too late and miss an invite.		1	
If we are re-applying or took a research year and have a mentor at a non-home institution, can we still get a letter from them?			
I would love to see them address how they plan on evaluating us effectively over a video interview, and to update us on their video interview plans, in general, to give us ample time to prepare.		1	
Clear communication regarding basic qualities of a typical applicant that is competitive for a given program. E.G report the average number of publications, Step scores, extracurricular involvement that historically has been commensurate with matched applicants at a given program. Would make it easier for students to gauge where they stand in terms of competitiveness for a given program.	1		
Ease your expectations, please! While many of us are extremely driven individuals that wanted to be saving the world during the covid pandemic, that just wasn't the reality. I have been quarantined XXX [personal situation] in a XXX [locale], and every volunteer opportunity I apply for never becomes anything. It is extremely frustrating, because I know my colleagues in the big cities are able to help with testing, contact tracing, supply delivery, etc. My point is, everyone's quarantine looks a little different. And to expect us medical students to have found some way to look awesome and have made this time incredibly productive compared to our colleagues is unrealistic.	1		
I think I am mostly just curious about how we can best express interest. I chose and applied to away rotations at universities to get "my foot in the door" as a student from a low-tier institution. I am more concerned about access to mid-high tier programs without away rotations and in-person interviews despite feeling like I have an at least "average" ENT application.			1
Have otolaryngology program directions considered conducting in-person second looks as a way to meet applicants and allow applicants to meet residents/faculty and see the facilities?			
What should students who have no home program be doing to show interest in programs with otu away rotations?			1

I think that an application cap will be mutually beneficial to students and programs. Programs will know that students applying to their institution have an actual interest, and students will not have the extreme cost of applying to a huge number of programs. Additionally, I worry that with virtual interviews "top applicants" will take a larger proportion of the available interview slots than they previously have, which will be bad for programs and other applicants alike. As a student without a home program, there have been XXX [comments] from academic otolaryngologists, but it would be nice to see something official saying that programs should be making an effort to "adopt" geographically close "orphan" students because most of the programs near me are just refusing outright to accept students, making it very unlikely that I will get to complete any Sub-I.		1	
Number of letters required given aways are no longer happening	1		
Clearly defined interview commitments, ie: would it be possible to have more than one program's interview in a single day. Do programs expect more than one day of availability (ie: both friday and saturday) when accepting the offer.		1	
What's the best way to show interest in a program in a year where programs will likely favor their home students?			1
A clear statement about requirements for the upcoming application cycle. Eg, make it 100% clear that letters can only come from home institution, etc.	1		
How are schools trying to keep the OTO applicant pipeline beyond this next match cycle? There are fewer opportunities for early OTO clinical exposure with current social distancing restrictions.			
They need to be more forgiving overall. Research is hard to come by and new relationships are almost impossible to make.			
Will programs interview the same number of applicants?		1	
XXX [Comment regarding concern for changed sub-I evaluations being misinterpreted by program directors]			
Will there be 2nd look weekends before Rank order lists are due?			
I am concerned that some students / programs may take the AAMC statement and the Otolaryngology PDs' statement too loosely and will still attend away rotations. There has been discussion amongst schools in XXX [city] or other large cities with multiple programs of doing local away rotations, which would just further disadvantage certain applicants. I am concerned that the statements were not strong enough and that some students will somehow try to game the system and get a leg up over others and in doing so potentially jeopardize the health of colleagues and patients.			
I'm concerned about the limitation of rec letters to just the home institution. If a student were to do a research year or a third year elective at another institution, they should be allowed to use their letter from that program			
How will having or not having Step 2 CK for applicants with a below average Step 1 score for ENT affect their application?	1		
