# Supplementary figures and images for: Otolaryngology Match 2020-21: Survey of Prospective Applicants in the Setting of COVID-19
Source: Ann Otol Rhinol Laryngol. 2020 Aug 19;130(5):450–8. doi: 10.1177/0003489420952470 (PMC7481654; doi:10.1177/0003489420952470)

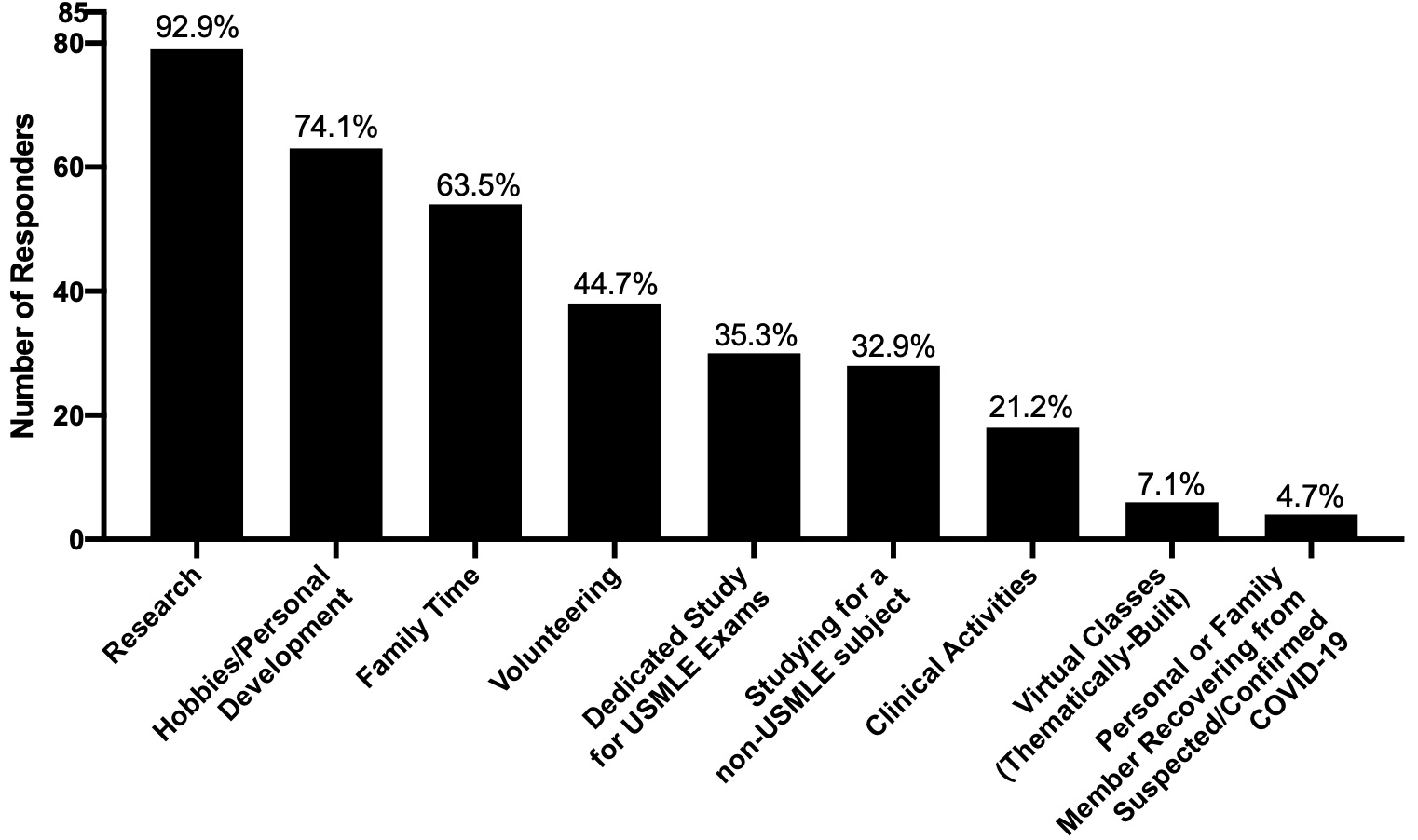

Supplement: Supplementary_Figure_3 – Supplemental material for Otolaryngology Match 2020-21: Survey of Prospective Applicants in the Setting of COVID-19 [file Supplementary_Figure_3.jpg]

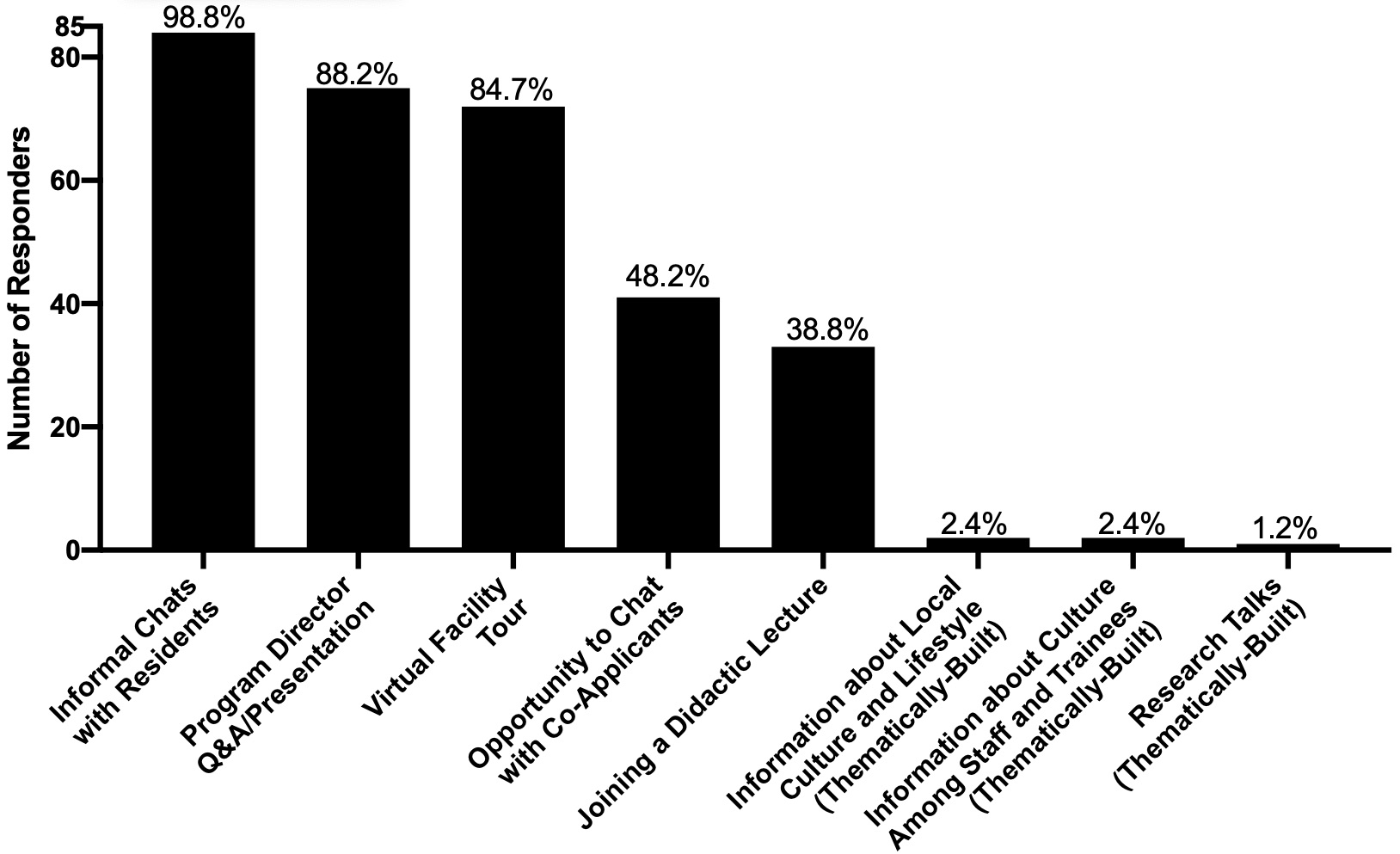

Supplement: Supplementary_Figure_4 – Supplemental material for Otolaryngology Match 2020-21: Survey of Prospective Applicants in the Setting of COVID-19 [file Supplementary_Figure_4.jpg]
